# Bupropion-disguised chest pain presenting in a middle-aged male: a case report and review of literature

**DOI:** 10.1097/MS9.0000000000002175

**Published:** 2024-05-28

**Authors:** Hasaan Ahmed, Mahmoud Ismayl, Anirudh Palicherla, Mohammad Selim, Ahmed Aboeata

**Affiliations:** a Department of Medicine, Division of Internal Medicine, Creighton University School of Medicine, Omaha, NE; b Division of Cardiovascular Disease, Department of Medicine, Creighton University School of Medicine, Omaha, NE; c Department of Cardiovascular Medicine, Mayo Clinic, Rochester, MN; d Department of Pulmonary and Critical Care, Loyola University Medical Center, Chicago, IL, USA

**Keywords:** bupropion, case report, chest pain, pharmacology

## Abstract

**Introduction and importance:**

Chest pain is one of the most prevalent complaints amongst individuals presenting in healthcare settings, encompassing a broad spectrum of etiologies. Work-up for chest pain often focuses on excluding life-threatening conditions before the consideration of atypical causes.

**Case presentation:**

A 47-year-old male with a past medical history of tobacco use and depression presented with persistent left-sided chest pain. Vitals on arrival were notable for mild hypertension. Two consecutive high-sensitivity troponins were unremarkable. The electrocardiogram showed sinus rhythm with no ischemic changes. Due to the atypical presentation of chest pain, the patient’s home medications were reviewed, and his bupropion was discontinued due to concern for medication-induced chest pain. The patient was discharged and presented 2 days for follow-up endorsing complete resolution of his chest pain.

**Clinical discussion:**

Prior investigations have shown bupropion to be associated with chest pain, with resolution noted after discontinuation. The etiology of chest pain is likely sympathomimetic, as bupropion has been shown to exhibit positive inotropic effects on myocardial tissue, propagated by catecholamine release.

**Conclusion:**

Patients taking bupropion may present with atypical chest pain. Medication discontinuation may be beneficial in alleviating symptoms.

## Introduction

Highlights

- Chest pain is a common complaint encountered by healthcare providers.
- Medications are often overlooked as causes of chest pain.
- Bupropion may cause chest pain due to its sympathomimetic properties.

Chest pain continues to be one of the most prevalent complaints encountered by healthcare providers, accounting for more than 6.5 million emergency department visits and 4 million outpatient visits annually^1^. The broad spectrum of differential diagnoses encompassing chest pain creates a diagnostic challenge, as providers must often navigate the vague symptoms endorsed by their patients while excluding life-threatening causes^2^. While the use of modalities, such as electrocardiograms and troponin markers, are important in evaluating chest pain, these tools are not definitive, as guidelines by the American College of Cardiology/ American Heart Association state that a comprehensive history is essential when evaluating these patients^1^.

Medications are an infrequent, but often overlooked, cause of chest pain. Buproprion is frequently prescribed by providers, with nearly 30 000 prescriptions annually in the United States^3^. Despite the widespread use of bupropion, there remains a significant gap in the understanding of bupropion’s effects on cardiovascular dynamics, particularly in propagating chest pain. We describe a rare case of chest pain presenting in a middle-aged male who was prescribed bupropion. Our case, which has been reported in line with SCARE (Surgical CAse REport) guidelines, aims to review the current literature of chest pain with bupropion use^4^.

## Case presentation

A 47-year-old male with a BMI of 41.3 and a past medical history of depression presented to the university hospital’s emergency department due to recurrent chest pain. The patient endorsed having three months of recurrent chest pain that was substernal in nature, occurring during both rest and with exertion. He stated that his chest pain occurred randomly without any precipitating or relieving factors. He also endorses intermittent episodes of radiating neck pain with his chest pain. His symptoms neither worsened nor improved with positional changes. The patient was a former smoker (1 and a half packs daily for 15 years) and had quit smoking one year before hospitalization. He denied the use of any illicit or recreational drugs. He did endorse drinking alcohol socially. He had no prior history of hospitalizations or surgeries. He had no personal or family history of heart disease. He also had no allergies.

The patient had been prescribed extended-release bupropion 150 MG to manage his depression symptoms about 4 months before presenting to the emergency room. About 1 month after starting bupropion, the patient endorsed having substernal chest pain that radiated to his neck and left arm. Due to his symptoms combined with his risk factors of obesity and being a former smoker, he underwent an exercise stress echocardiogram, which showed no stress-induced wall motion abnormalities. The patient’s chest pain was attributed to be non-cardiac in etiology, and he was told to monitor his symptoms.

**Figure 1:**
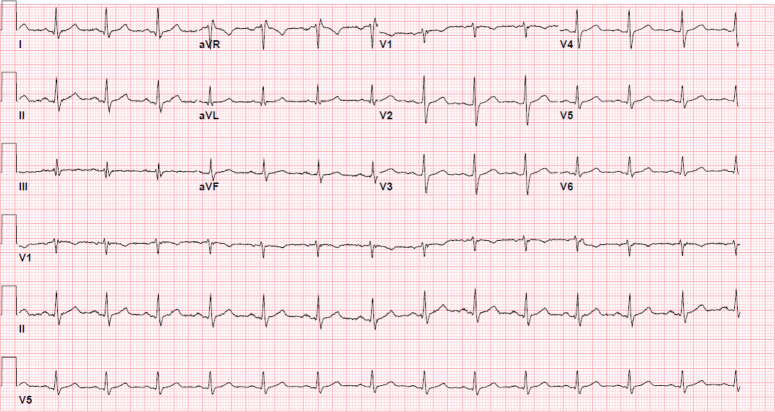
Electrocardiogram showing no findings to suggest ischemia.

**Figure 2:**
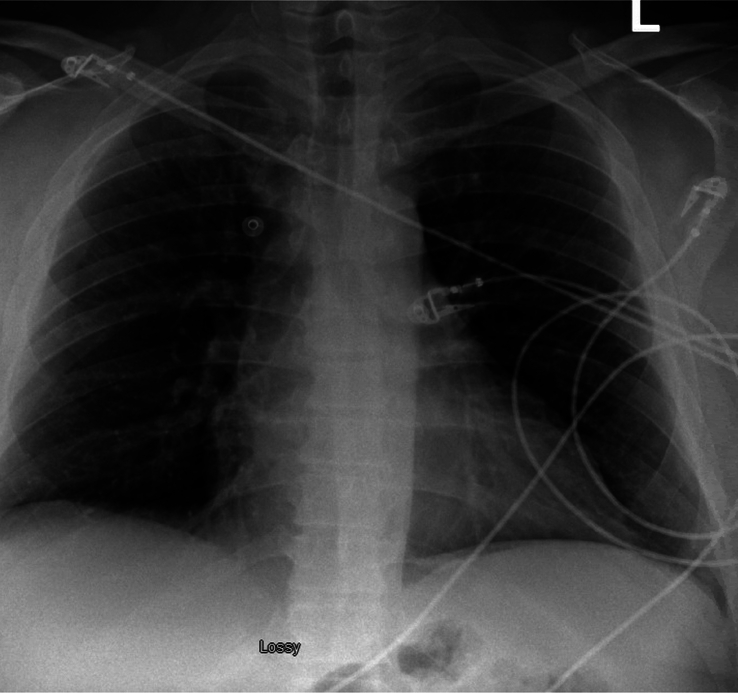
Chest X-ray negative for focal airspace consolidation, pleural effusion, or pneumothorax.

**Figure 3:**
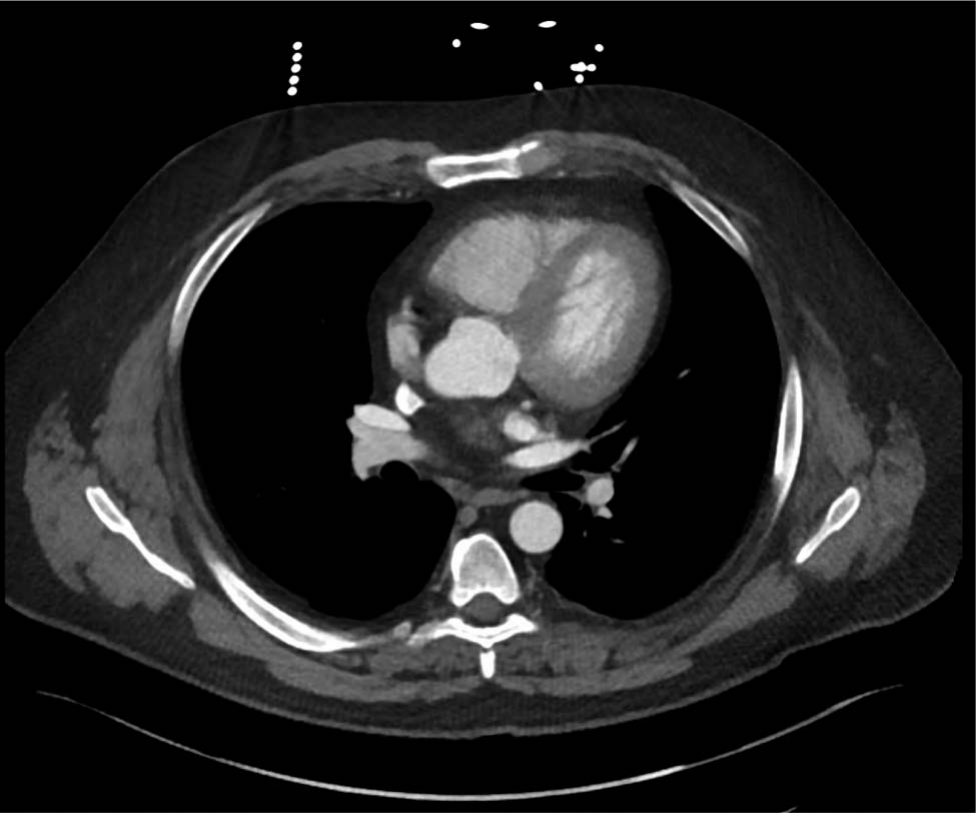
Computed tomography angiography of the chest showing no evidence of aortic dissection.

Vitals on admission were notable for hypertension (175/110) but were otherwise unremarkable. There were no financial or cultural barriers in evaluating the patient. Electrocardiogram showed the patient to be in sinus rhythm with no findings to suggest ischemia (Fig. 1). Chest X-ray showed no focal airspace consolidation, pleural effusion, or measurable pneumothorax (Fig. 2). Initial high-sensitivity troponin (16.3; reference ≤77.0 ng/l) and repeat high-sensitivity troponin (14.8; reference ≤77.0 ng/l) markers were both unremarkable. Complete blood cell count and B-type natriuretic peptide (100; reference ≤124 pg/ml) were also noncontributory. Due to the patient’s chest pain in the setting of hypertension, computed tomography angiography (CTA) of the chest was obtained, which was negative for aortic dissection (Fig. 3).

In the emergency department, the patient received intravenous aspirin 324 mg due to the and morphine 2 mg due to the initial concern for acute coronary syndrome. However, his chest pain minimally improved, Subsequently, the patient was admitted for observation and further evaluation.

Differential diagnoses included acute coronary syndrome, aortic dissection, gastroesophageal reflux disease, and pericarditis. Throughout the patient’s hospitalization, his chart and history were extensively reviewed to further evaluate the context of his chest pain. Given the onset of his chest pain after being prescribed bupropion, the decision was made to hold it while hospitalized. The patient began to slowly endorse improvement of his chest pain, and the decision was ultimately made to discontinue his home bupropion on discharge and start the patient on sertraline 25 mg daily to manage his depression. The patient was discharged on day four of hospitalization and was advised to abstain from taking bupropion. During his primary care follow-up one week later, he endorsed having significant resolution of his chest pain and gratitude for the care he received in the setting of continued abstinence from bupropion. The patient had no further hospitalizations or acute-care visits complaining of chest pain. During his annual physical exam with his primary care provider 6 months later, he stated he was doing well overall with no further episodes of chest pain.

## Discussion

Bupropion is a commonly prescribed medication and is often used for depression, smoking cessation, attention-deficit/hyperactivity disorder, and seasonal affective disorder^5^. While the specifics of bupropion’s pharmacological mechanisms remain not fully understood, It is thought that bupropion inhibits the reuptake of both dopamine and norepinephrine, with a limited effect on serotonin reuptake^5,6^. This has been affirmed in several clinical human studies, which noted a reduction in norepinephrine degradation with bupropion administration, suggesting that bupropion exhibits profound noradrenergic properties Bupropion exhibits sympathomimetic properties, which is likely attributed to its similar cellular structure with amphetamines, as they both contain a phenylethylamine backbone^7^.

While the mechanism of chest pain in bupropion is not fully understood, we hypothesize that it may be driven by increased myocardial oxygen demand, propogated by the release of catecholamines^8^. The impact of bupropion on catecholamine release was explored in an investigational study by Cremers *et al.* ^9^, who noted that increased concentrations of bupropion were associated with a positive inotropic response in human cardiac tissue (*P*<0.05). The increased release of catecholamines is likely driven by bupropion’s sympathomimetic properties, as evident by its amphetamine-like molecular composition^9^. Other disturbances in cardiovascular dynamics have been noted with bupropion use, including hypotension, hypertension, and orthostatic changes^9^. This is evident in an investigational study by Roose and colleagues, who found that bupropion was associated with an increase in supine systolic blood pressure by 5 mmHg (*P*<0.01), an increase in supine diastolic blood pressure by 3 mmHg (*P*<0.005), and an increase in orthostatic blood hypotension by 3 mmHg (*P*<0.02)^10,11^. Furthermore, ventricular premature depolarization complexes were noted to be significantly repressed with bupropion use (*P*<0.005)^10,11^. The disturbance in hemodynamic physiological processes, propagated by bupropion, may have amplified the chest discomfort our patient was experiencing.

Prior studies evaluating chest pain with bupropion use remain limited. A study by De Graaf *et al.* ^12^ investigated chest complaints associated with bupropion use in which out of 591 individuals prescribed bupropion, 22 endorsed having a sensation of chest pain or chest pressure. Furthermore, this association of bupropion with chest pain was noted to be statistically significant [odds ratio (OR) 0.25; 95% CI 4.97–13.68], with the average onset for chest pain noted to be 6 days^12^. In a randomized controlled trial evaluating the role of bupropion for smoking cessation by Jorenby *et al*.^13^, 11.9% of study participants stopped using bupropion due to adverse effects, with discontinuation rates noted to be significantly higher among those administered bupropion compared to those who received a placebo (*P*=0.004). Similar to our patient, there was one study participant who endorsed having chest tightness and dyspnea, which was ultimately relieved with bupropion discontinuation; however, the statistical significance was not assessed^13^. Further studies are warranted to investigate the impact of bupropion on inciting chest pain.

Previous case reports have also noted chest pain with bupropion use. A case by Patterson *et al.* ^14^ described acute myocardial infarction in a patient who developed chest pain 2 weeks after starting bupropion, with medication-induced coronary artery vasospasm as a potential explanation. Similarly, Serafini *et al.* ^15^ reported a case of chest pain occurring in a patient whose bupropion was up-titrated to 300 mg daily, which later improved with dose-reduction, with the patient’s chest pain also attributed to drug-induced coronary vasospasm. However, both these case reports are limited by their hypothetical assumptions that chest pain was a product of bupropion use. Nonetheless, adverse effects associated with bupropion remain a concern, as prospective studies have been used to assess its pharmacologic safety profile^14^. While the association between bupropion and chest pain may be circumstantial, large-scale investigations of chest pain with bupropion are needed^14^.

Although we hypothesize that our patient’s chest pain was a result of bupropion use, it’s important to discuss potential biases as well as alternative explanations involved in the work-up of our patient’s symptoms. Given the fact that our patient presented with the typical symptoms of acute coronary syndrome, combined with his risk factors of smoking, anchoring bias was likely involved as the patient’s chest pain was thought to be ischemic in etiology. Furthermore, alternative explanations for our patient’s symptoms include psychological factors, such as anxiety and stress, as well as somatic elements.

Our case report encompasses several limitations and strengths. One limitation is that being this is a case report, there is no control group, and therefore, we are not able to assess the impact of bupropion on inciting chest pain compared to a placebo. Another limitation is the verifiability of whether our patient’s symptoms were truly due to bupropion, or other causes such as musculoskeletal or psychological ailments. The fact that our patient’s chest pain resolved with cessation of bupropion neither necessarily imply that all patients taking bupropion should undergo routine screening for chest pain nor does it imply that bupropion should be contraindicated in patients with cardiovascular disease. Rather, our case aims to highlight the need for further studies into evaluating the effects of bupropion on cardiovascular dynamics and inciting chest pain. The strength of our case report encompasses the educational awareness it brings into highlighting that most cases of chest pain are non-cardiac in etiology. Furthermore, our case report emphasizes the need for providers to take both the clinical context as well as a comprehensive history of their patient into consideration when assessing chest pain.

## Conclusion

Chest pain is a frequent complaint encountered by providers, encompassing a broad spectrum of causes. Our case of chest pain presenting in a middle-aged male taking bupropion expands upon the current literature of adverse effects associated with bupropion use. This case report sheds light on the importance of comprehensive and routine monitoring in patients prescribed bupropion by providers, especially inquiring about potential cardiac complications such as chest pain. Furthermore, our case emphasizes the importance of providers using a holistic approach when assessing chest pain, taking into account the context of the patient’s medical history, social history, medication use, and cardiovascular risk factors.

## Ethical approval

No ethics approval was required for this manuscript.

## Consent

Written informed consent was obtained from the patient for publication of this case report and accompanying images. A copy of the written consent is available for review by the Editor-in-Chief of this journal on request.

## Source of funding

No funding was sought or utilized for this manuscript.

## Author contribution

Conceptualization: H.A., M.I., A.P., M.S., A.A. Writing—original draft: H.A., M.I., A.P. Writing—review and editing: H.A., M.I., A.P., M.S., A.A. Investigation: H.A., M.I., A.P. Supervision: M.S., A.A.

## Conflicts of interest disclosure

The authors declare that they have no known competing financial interests or personal relationships that could have appeared to influence the work reported in this paper.

## Research registration unique identifying number (UIN)

Not applicable.

## Guarantor

Hasaan Ahmed.

## Data availability statement

We, the authors, have nothing to declare in this category as it is not applicable.

## Provenance and peer review

Not commissioned, externally peer-reviewed.
